# Safety and feasibility of laparoscopy technology in right hemihepatectomy

**DOI:** 10.1038/s41598-019-52694-5

**Published:** 2019-12-11

**Authors:** Xin Yu, Dilai Luo, Yupeng Tang, Mingwen Huang, Yong Huang

**Affiliations:** 1 Department of General Surgery, the Second Affiliated Hospital of Nanchang University, Nanchang, 330006 China; 2 Department of Hepatobiliary Surgery, Mengchao Hepatobiliary Hospital of Fujian Medical University, Fuzhou, 350025 China

**Keywords:** Gastrointestinal system, Hepatology

## Abstract

Laparoscopic hepatectomy (LH) has been accepted widely owing to its advantages as a minimally invasive surgery; however, laparoscopic right hemihepatectomy (LRH) has rarely been reported. We aimed to compare the benefits and drawbacks of LRH and open approaches. Between January 2014 and October 2017, 85 patients with tumor and hepatolithiasis who underwent LRH (*n = *30) and open right hemihepatectomy (ORH) (*n = *55) were enrolled in this study. For tumors, LRH showed significantly better results with respect to blood loss (*P* = 0.024) and duration of hospital stay (*P* = 0.008) than ORH, while hospital expenses (*P* = 0.031) and bile leakage rate (*P* = 0.012) were higher with LRH. However, the operative time and rate of other complications were not significantly different between the two groups. However, for hepatolithiasis, there was less blood loss (*P* = 0.015) and longer operative time (*P* = 0.036) with LRH than with ORH. There were no significant difference between LRH and ORH in terms of hospital stay, hospital expenses, and complication rate (*P* > 0.05). Moreover, the postoperative white blood cell count, alanine aminotransferase level, aspartate aminotransferase level, and total bilirubin were not significantly different in both types of patients (*P* > 0.05). Our results suggest the safety and feasibility of laparoscopy technology for right hemihepatectomy in both tumor and hepatolithiasis patients.

## Introduction

With the recent advances in technique and equipment in laparoscopic technology, several comparative series have suggested the feasibility and safety of laparoscopic hepatectomy (LH)^1–10^, including minor liver resections^1–4^, left hepatic lobe^5,6^ major hepatectomies^1,2,7,8^, and left hemihepatectomy^9–11^. Our previous research also showed that laparoscopic left lateral hepatic sectionectomy^6^ and laparoscopic left hemihepatectomy^11^ offered significantly more advantages than open surgery. However, thus far, few studies have explored the details of laparoscopic right hemihepatectomy (LRH). The right hemiliver exhibits a relatively complex intrahepatic tract. More importantly, the right liver is located deep in the abdominal cavity, and mobilization and exposure remain difficult due to the lack of a smooth and safe liver retractor^12,13^. These anatomical features of the right liver present an unfavorable condition for laparoscopy. Thus, till date, there was insufficient research to summarize the benefits and drawbacks of LRH compared with those of ORH. Moreover, there is no unified conclusion about the optimal approach, and no clear guidelines exist for the indications of the laparoscopic approach. The present study aimed to compare the clinical and economic impact of LRH and ORH in both benign and malignant lesions.

## Methods

### Patients and grouping

This retrospective clinical study was conducted at the Department of General Surgery and approved by the institutional review board (IRB) committee at the Second Affiliated Hospital of Nanchang University. Informed consent was obtained from all the subjects. This study was performed as per the established national and institutional ethical guidelines regarding the involvement of human subjects and the use of human tissues for research. All the patients with tumor and hepatolithiasis (Table 1) who underwent LRH (*n = *30) or ORH (*n = *55) were enrolled from the database between January 2014 and July 2017. The study was approved by the Second Affiliated Hospital of Nanchang University Ethics Committee, and specimens were collected after obtaining informed consent from the patients.

**Table 1:** Patient characteristics.

	LRH (*n* = 30)	ORH (*n* = 55)	*P*
Gender (M/F)	16/14	31/24	0.989
Age (years)	59 ± 16	61 ± 13	0.548
**Laboratory data**			
WBC (10^9^/L)	12.5 ± 4.8	13.5 ± 4.4	0.315
AST (U/L)	212(117,276)	221(140,474)	0.312
ALT (U/L)	158(83,238)	186(106,375)	0.081
Operative time (min)	312 ± 79	273 ± 72	**0.023**
Blood loss (ml)	341 ± 121	552 ± 323	**0.001**
Transfusion	14	36	0.093
Hospital stay (days)	11.5 ± 3.4	14.5 ± 4.5	**0.002**
Hospital expenses (WanRMB)	5.6 ± 1.6	5.0 ± 1.5	0.088
Conversion		—	
**Postoperative complication**			
Hemorrhage	1	1	0.622
Intraabdominal fluid collection	6	13	0.702
Wound complication	1	8	0.110
Bile leakage	8	5	**0.031**
Heart failure	0	1	0.460
Mortality	0	1	0.460

WBC, blood cell count; AST, aspartate aminotransferase; ALT, alanine aminotransferase; WanRMB, ten thousand renminbi.

### LRH and ORH procedure

**Figure 1:**
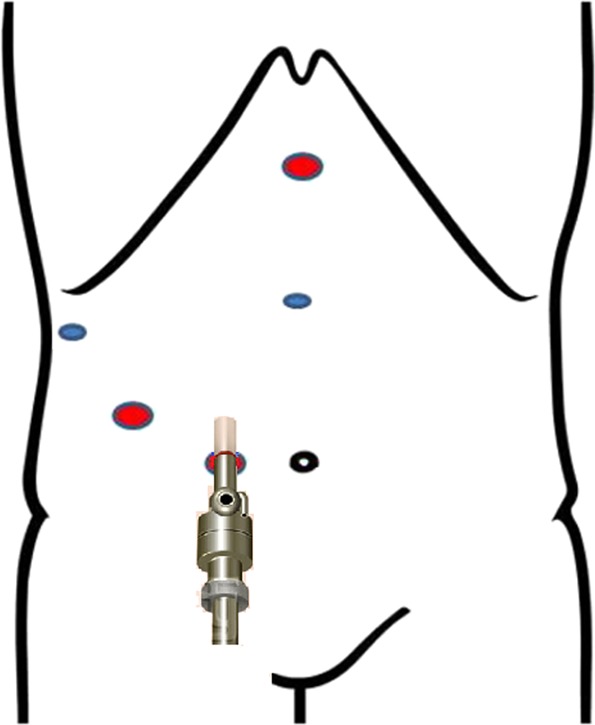
The position of the trocars.

**Figure 2:**
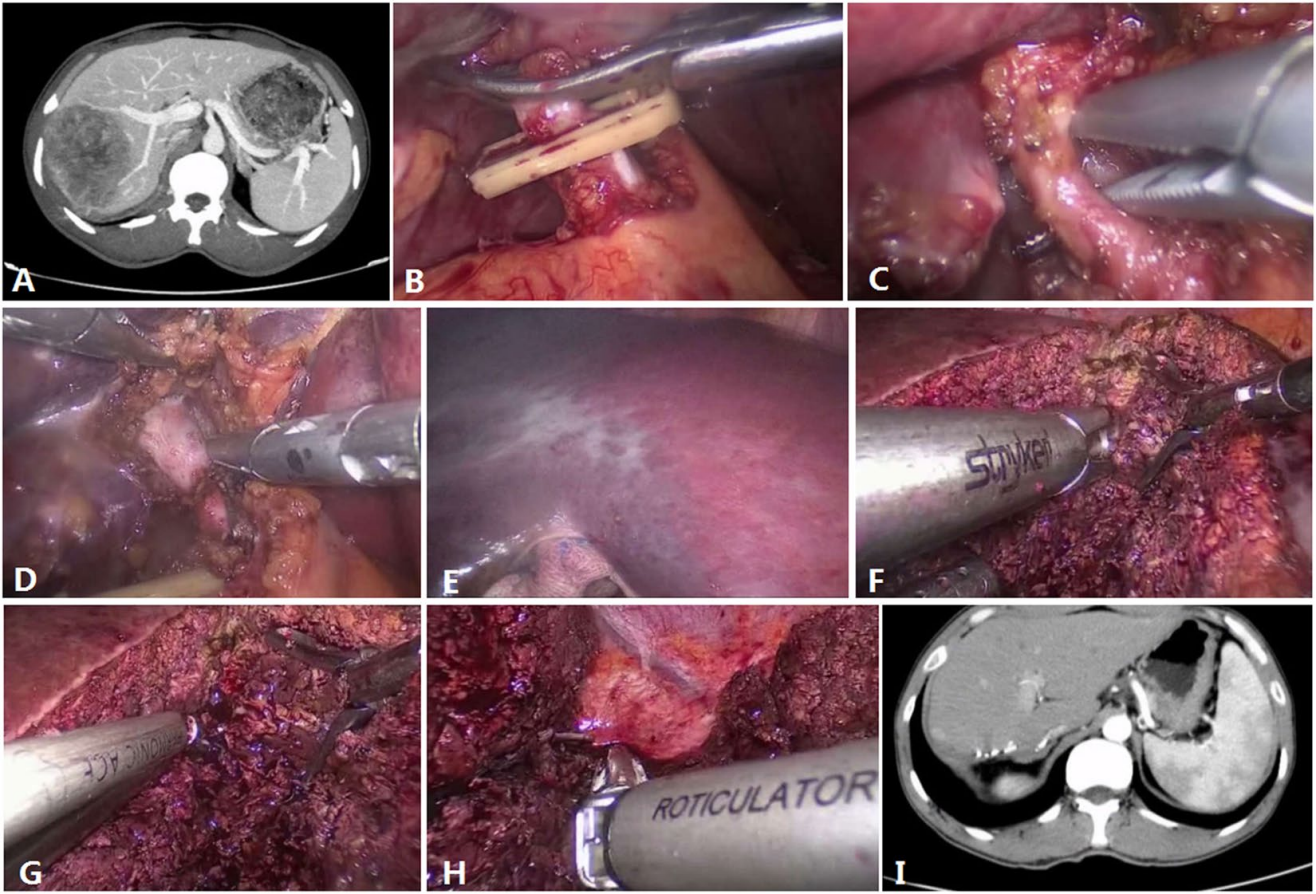
Surgical techniques for LRH. (**A**) the preoperative CT film of patient; (**B**) The cystic duct was dissected and ligated; (**C**) The right hepatic artery were dissected and then ligated. (**D**) The right portal vein was dissected and was ligated. (**E**) The hepatic ischemic line; the left intrahepatic bile ducts are interrupted and the visible stones are removed. (**F**,**G**) Intrahepatic duct isolated and ligated; (**H**) The right hepatic vein and the surrounding parenchymal tissues are transected using an Endo-GIA device. (**I**) The CT film of patient after 1 month.

Under general anesthesia, the patient was placed in the supine position with both the legs separated. A 12-mm trocar was placed on the right side of the umbilical cord 2 cm away for laparoscopy. The other trocars were positioned appropriately in the upper abdomen to allow optimal mobilization and dissection of the liver, as shown in Fig. 1. The two main operating 12-mm trocars were located below the xiphoid and 5 cm below the midline of the right clavicle. The other two 5 mm trocars were placed 2 cm above the umbilical cord and under the subcostal at the anterior axillary line respectively. The patient was tilted 30°–45° to the left. The pneumoperitoneum was established by maintaining an intra-peritoneal pressure of 12–14 mmHg and differential pressure with the central venous pressure (CVP) of 4–6 mmHg. The liver anatomy and the right liver were evaluated using laparoscopy. In addition, laparoscopic liver ultrasonography was used to determine the location and extent of the lesions and identify potentially hazardous intrahepatic vascular and biliary structures. The right hepatic artery were first dissected and then ligated with a Hem-o-Lok clip. The right portal vein was dissected and was ligated using Hem-o-Lok clips. We distinguish the left from the right hemiliver based on the hepatic ischemic line; however, the middle hepatic vein is occasionally damaged due to deviation. Therefore, it is crucial to determine the direction of the middle hepatic vein before dissection. We should find the branch of the middle hepatic vein first and then look for the trunk along the branch. Laparoscopic ultrasonography ensures that the middle hepatic vein is completely avoided while providing images of intrahepatic intubation^14^. We ligated intrahepatic vascular or biliary using Hem-o-Lok clips. The right hepatic vein was ligated using the Hem-o-Lok clips or the Endo-GIA stapler. The specific steps are shown in Fig. 2. The steps of ORH were similar to those of LRH.

### Statistical analyses

Statistical analyses were performed with SPSS software, version 17.0 (SPSS Inc., Chicago, IL, USA). Continuous variables are expressed as mean ± standard deviation or median (range) values, and categorical variables are expressed as numbers. Continuous variables were compared using Student’s *t*-test test or the Mann–Whitney *U*-test, while categorical variables were compared using the *χ*^2^ test or Fisher’s exact test. *P < *0.05 was considered statistically significant.

## Results

### Patient characteristics

Total 85 patients who underwent LRH (*n* = 30) or ORH (*n* = 55) were enrolled between January 2014 and July 2017. Their demographic and clinical/laboratory data are presented in Table 1. There were no significant inter-group differences in terms of sec, age, and liver function (*P* > 0.05). Of the 30 patients in the laparoscopic group, 18 had hepatolithiasis and 12 had tumor. Of the 55 patients in the open group, 38 had hepatolithiasis and 17 had tumor.

### Perioperative outcome

The surgical outcomes have been shown in Table 1. LRH patients had longer operative times (*P = *0.023) and higher bile leakage (*P = *0.031); however, they had less blood loss (*P = *0.001) and shorter hospital stay (*P = *0.002) than the ORH patients. Furthermore, there were no significant differences in the frequency and intensity of other complications, postoperative white blood cell (WBC) count, aspartate aminotransferase (AST) level, and alanine aminotransferase (ALT) level (*P* > 0.05), suggesting no difference in the perioperative liver injury or functional outcome.

We further divided the patients into the tumor and hepatolithiasis group. Among the tumor group, 12 patients were in the LRH group, and 17 were in the ORH group. With respect to the hepatolithiasis group, 18 patients were in the LRH group, and 38 were in the ORH group. As shown in Table 2, the LRH showed significantly better results in terms of less blood loss (*P* = 0.024) and shorter hospital stay (*P* = 0.008); however, LRH patients also incurred a lower hospital cost (*P* = 0.031) and had a higher bile leakage rate (*P* = 0.012) than ORH patients. Moreover, there was less blood loss (*P* = 0.015) and longer operative time (*P* = 0.036) for LRH than for ORH, as shown in Table 3. However, there were no statistical differences between LRH and ORH in terms of the hospital stay; hospital expenses; complication rate; as well as the postoperative WBC, AST, and ALT levels (*P* > 0.05) in both the tumor and hepatolithiasis group.

**Table 2:** Characteristics of the tumor patients.

	LRH(*n* = 12)	ORH (*n* = 17)	*P*
Gender (M/F)	10/8	21/17	0.372
Age (years)	59 ± 17	61 ± 14	0.785
Cirrhosis	5	9	0.550
Primary liver cancer	9	12	0.793
Tumor size (cm)	4.9 ± 1.1	5.1 ± 1.3	0.341
**Laboratory data**			
WBC (10^9^/L)	11.6 ± 4.5	14.9 ± 5.2	0.089
AST (U/L)	160(82,264)	136(69,221)	0.188
ALT (U/L)	117(53,190)	126(78,236)	0.145
Operative time (min)	289 ± 74	260 ± 67	0.281
Blood loss (ml)	315 ± 131	506 ± 253	**0.024**
Transfusion	5	11	0.219
Hospital stay (days)	9.8 ± 1.8	13.4 ± 4.2	**0.008**
Hospital expenses (WanRMB)	5.4 ± 2.5	4.7 ± 2.3	**0.031**
**Postoperative complication**			
Hemorrhage	0	0	—
Intraabdominal fluid collection	2	4	0.659
Wound complication	0	1	0.401
Bile leakage	4	0	**0.012**
Mortality	0	0	—

WBC, blood cell count; AST, aspartate aminotransferase; ALT, alanine aminotransferase; WanRMB, ten thousand renminbi.

**Table 3:** Characteristics and perioperative outcomes of the hepatolithiasis patients.

	LRH (*n* = 18)	ORH (*n* = 38)	*P*
Gender (M:F)	10/8	21/17	0.974
Age (years)	57 ± 14	59 ± 11	0.644
**Laboratory data**			
WBC (10^9^/L)	13 ± 5	12.9 ± 3.9	0.906
AST (U/L)	252 (131,295)	252 (171,541)	0.790
ALT (U/L)	166 (107,244)	232 (132,436)	0.452
Operative time (min)	328 ± 80	279 ± 75	**0.036**
Blood loss (ml)	359 ± 113	572 ± 351	**0.015**
Transfusion	9	25	0.259
Hospital stay (days)	12.7 ± 3.7	14.9 ± 4.6	0.082
Hospital expenses (WanRMB)	5.7 ± 0.8	5.2 ± 0.9	0.395
**Postoperative complication**			
Hemorrhage	1	1	0.585
Intraabdominal fluid collection	4	9	0.905
Wound complication	1	7	0.203
Bile leakage	4	5	0.393
Residual stones	5	5	0.186
Mortality	0	1	0.491

WBC, blood cell count; AST, aspartate aminotransferase; ALT, alanine aminotransferase; WanRMB, ten thousand renminbi.

## Discussion

LH has matured and become a treatment option for benign and malignant lesions of the liver^1–10^, especially for left hepatectomy^5,6,9–11^. However, few studies have focused on LRH owing to the anatomical features^12,13^. It was difficult to mobilize and expose, and it was also difficult to control bleeding by laparoscopy. In this study, we aimed to compare the clinical and economic impact of LRH and ORH in patients with tumors and hepatolithiasis. Our results suggest that LRH offers several advantages over ORH, including reduced blood loss, lower hospital stay, and fewer complications.

Our results show that LRH is associated with a longer operative time, but less blood loss and shorter hospital stay than ORH. For longer operative time and shorter hospital stay, our results were consistent with previous research^2,12,15,16^. However, bleeding control during LRH was superior to that during open hepatectomy, possibly due to improved intraoperative magnification for surgical manipulations, use of new coagulating devices unlike expected, and the pressure associated with the pneumoperitoneum that may have helped decrease bleeding during liver parenchymal transection^4,12,17,18^. We believe that occasional bleeding and hepatic vein injury are the most common hepatectomy risks, irrespective of which approach is selected for use. Hence, detailed preoperative evaluations, including computed tomography, magnetic resonance imaging, 3D visualization technology, and especially intraoperative ultrasonography are essential to accurately reveal the size and location of the lesion and understand the individual variations in the blood vessels and the biliary tract in order to reduce the bleeding risk. Given the use of different surgical procedures and complexity, we further divided the patients into the tumor group and the hepatolithiasis group. For tumor patients, the operative time in the LRH group was similar to that in the ORH group; however, the LRH group experienced less blood loss and shorter hospital stay with higher hospitalization costs than the ORH group. As per our understanding, the rapid developments and advances in laparoscopic equipment, such as the Endo-GIA stapler, not only greatly reduce the operative time but also effectively prevent bleeding^19^. Most importantly, the surgeon’s effective and professional knowledge have improved their technical skills over time and made them more confident for performing surgery, reflecting the standardization of the surgical procedures. With respect to the hospitalization costs, LRH was associated with significantly higher costs than ORH. The laparoscopic material, such as laparoscopy trocars, Hem-o-Lok clips, and staplers, along with the longer operative time contributed to the increased hospital costs of LRH^13,15,20^. Moreover, ultrasonic shears, vessel sealing devices (e.g., LigaSure), laparoscopic ultrasonography systems, and suturing techniques are being improved constantly, reducing the operative time while increasing the cost. The surgical procedure for hepatolithiasis is different from that for a tumor and is therefore more challenging. Severe perihepatic adhesions, deformed biliary anatomy, and fibrotic parenchyma in patients with hepatolithiasis may not only prolong the operation time, but also may increase the risk of postoperative complications, such as intraabdominal fluid collection and biliary fistula^21^. Considerable time is required to dissect the adhesions caused by inflammation around the liver and remove the stones from the bile duct in hepatolithiasis patients. Sometimes, choledochoscopy and hydroelectric lithotripsy need to be performed. In most cases, a T tube needs to be placed. These additional operations were not only cumbersome and complicated, but also lacked the advantages offered by laparotomy, such as operating horizon and space and tactile feedback; in addition, they require greater hand-eye coordination^22^. So, it was not surprising. Consistent with these reports, our results showed that the hepatolithiasis patients in our study who underwent LRH had longer operative times and less blood loss than those who underwent ORH^23,24^. Although LRH had no obvious superiority in terms of the operative time and hospital stay, it still had the advantage of less bleeding. Furthermore, the two groups showed no significant difference in the postoperative alanine transferase, albumin, and total bilirubin levels, suggesting no difference in the extent of perioperative liver injury or functional outcome.

With respect to the complications, our results showed that the tumor patients in our study who underwent LRH demonstrated a higher bile leakage rate than those who underwent ORH; however, no such difference was observed for the hepatolithiasis patients. Other complications were comparable between the two groups. It was partially consistent with laparoscopic left hepatectomy^9,11^. The most important reason was that we routinely performed water injection experiments from the cystic tube to detect the presence of bile leakage in tumor patients who underwent ORH but not in those who underwent LRH. The measure considerably reduced the probability of bile leakage. However, in case of hepatolithiasis patients, T tube drainage was performed regularly for both LRH and ORH patients^25^.

For LRH, we recommend that the patient be tilted to the left so that the liver is turned to the left to facilitate exposure of the right liver due to gravity. The middle hepatic vein branch and the trunk along the branch should then be identified. In addition, CVP should be maintained between 4 and 6 cm H_2_O, the optimal intraoperative range for reducing bleeding and hepatic vein reflux^26^.

In conclusion, this comparative study suggests that laparoscopy is suitable for right hepatectomy. Our results show that it is safe and feasible to perform to use laparoscopy technology for right hemihepatectomy in both tumor and hepatolithiasis patients. It is expected to be the first choice for the treatment of right hemiliver.
